# Remotely Administered Walking Tests for Assessing Functional Capacity in Patients with Chronic Pulmonary Diseases or Heart Failure: A Systematic Review of Agreement, Reliability, Feasibility and Clinical Utility

**DOI:** 10.3390/healthcare14050576

**Published:** 2026-02-25

**Authors:** Eleni A. Kortianou, Maria Isakoglou, Eleni Karagianni, Varsamo Antoniou, Vaia Sapouna, Garyfallia Pepera

**Affiliations:** Clinical Exercise Physiology and Rehabilitation Laboratory, Physiotherapy Department, University of Thessaly, 35132 Lamia, Greece; misakoglou@uth.gr (M.I.); elkaragiann@uth.gr (E.K.); varsamoantoniou@uth.gr (V.A.); vsapouna@uth.gr (V.S.); gpepera@uth.gr (G.P.)

**Keywords:** chronic pulmonary diseases, agreement, feasibility, heart failure, reliability, utility, walking tests

## Abstract

**Highlights:**

**What are the main findings?**

- RaWTs in people with chronic pulmonary diseases or heart failure appear reliable, feasible, and acceptable to patients.
- Validity with in-clinic testing is context-dependent and strongly influenced by test setup and environmental conditions.

**What are the implications of the main findings?**

- The clinical utility of the remote testing is still limited.
- The remotely administered 6 min walk test can be used with minimal safety concerns when implemented under structured protocols as it demonstrates good to excellent test–retest reliability and high feasibility with strong patient adherence.

**Abstract:**

**Objective**: The objective of this study is to conduct a systematic review of the evidence on the use of remotely administered walking tests (RaWTs) in patients with chronic pulmonary diseases (CPDs) and heart failure (HF), focusing on agreement, reliability, feasibility, and clinical utility as outcomes. **Methods**: This study followed the Preferred Reporting Items for Systematic Reviews and was registered on the International Prospective Register of Systematic Reviews platform (ID: CRD420251180996). The PubMed, Web of Science, CENTRAL, Scopus, and ACM databases were comprehensively searched from inception up to October 2025. Observational, randomized and non-randomized control studies assessing the agreement, reliability, feasibility, and clinical utility of RaWTs in people with CPDs and HF and reporting quantitative outcomes were eligible. Two reviewers independently conducted study selection, data extraction, and risk of bias assessment using the COSMIN Risk of Bias tool for the reliability studies, the Risk of Bias in Non-Randomized Studies—of Interventions (ROBINS-I) tool for non-randomized studies, and the Quality in Prognosis Studies (QUIPS) tool for the prognostic studies. **Results**: Eleven studies met the inclusion criteria. Five studies included patients with HF, five with pulmonary hypertension (PH), and one study included candidates for lung transplantation due to advanced CPD. All studies used the 6 min walk test (6MWT); one also included the incremental shuttle walk test. Agreement with face-to-face in-clinic testing (in five studies) is setting-dependent and influenced by the testing setup. Reliability (in eight studies), derived from variable statistical indices in both patient populations, showed that RaWTs are reliable. Adherence and safety were used as the main feasibility indicators. Eight studies concluded that remote assessment is feasible, acceptable, and safe. Clinical utility was examined in only one HF study, showing that remotely administered 6MWT can predict all-cause mortality and HF hospitalization. According to COSMIN, the overall methodological quality of nine studies ranged from very good to inadequate. One study was rated as having a serious risk of bias according to ROBINS-I, and one study as having a high risk of bias according to QUIPS. **Conclusions**: Although the evidence is limited and heterogeneous, RaWTs demonstrate robust reliability across repeated measurements while agreement with in-clinic testing is context-dependent and strongly influenced by test setup and environmental conditions. RaWTs appear to be acceptable to patients; however, further high-quality studies are needed to confirm these findings and determine the clinical utility of RaWTs on specific clinical outcomes in these populations.

## 1. Introduction

Functional assessment constitutes a cornerstone in the clinical management of patients with chronic pulmonary and cardiovascular diseases, providing essential diagnostic insight, prognostic information, and guidance for therapeutic decision-making [1,2,3]. Historically, functional capacity assessment has been performed exclusively within controlled clinical environments, where the patient’s physical presence allows for standardized testing procedures and direct supervision by specialized healthcare professionals. This functional assessment captures performance under clinical-related settings and time-limited conditions, lacking sensitivity to day-to-day variability, whereas remote assessment can continuously quantify real-world functional behavior, capturing day-to-day variability and clinically meaningful limitations that may be missed during a single supervised visit [4,5], enabling longitudinal monitoring of functional capacity over time, and supporting earlier detection of clinically meaningful deterioration and early functional decline [5]. Remotely administered walking tests (RaWTs) provide benefits such as reducing the logistical burden for both patients and healthcare providers, decreasing the necessity for in-person clinic visits, and improving accessibility for individuals residing in rural or underserved regions, delivering long-term hybrid care [6]. They may also enable earlier detection of physiological deterioration when conducted at regular intervals [5].

Before the emergence of the COVID-19 pandemic, the literature on remotely administered functional tests—particularly among individuals with chronic pulmonary diseases (CPDs) or heart failure (HF) was limited and primarily confined to small-scale feasibility investigations [7,8]. The COVID-19 pandemic profoundly disrupted conventional healthcare delivery models due to mandated social distancing measures and the heightened clinical vulnerability of cardiopulmonary patient populations [9,10]. This context created an urgent need for continuous monitoring and ongoing rehabilitation, acting as a catalyst for the accelerated integration of telemedicine and tele-rehabilitation services [11]. Consequently, remote functional assessment rapidly evolved from an adjunct modality into an indispensable component of patient care, offering a safe, accessible, and pragmatic alternative to in-person evaluations [12,13].

Simultaneously, advances in digital health technologies have facilitated the broader deployment of remote assessment strategies [14]. The incorporation of telemonitoring systems, remotely supervised exercise interventions, and distance-based measurement tools enabled structured and systematic evaluation of functional capacity within patients’ homes and community settings [10]. These developments stimulated a substantial increase in global research activity, driving methodological refinement, technological innovation, and an expanding evidence base in selected contexts [11,14]. Wearable sensor-supported tele-rehabilitation has shown significant promise in improving functional capacity and exercise adherence in cardiac [10] and respiratory populations [15].

Walking-based functional tests—most notably the Six-Minute Walk Test (6MWT) and the Incremental Shuttle Walk Test (ISWT)—are fundamental for assessing exercise tolerance, symptom burden, and rehabilitation outcomes in individuals with chronic pulmonary diseases and heart failure [1,16]. Although traditionally performed under direct clinical supervision, these assessments have been successfully adapted for remote implementation through the use of digital technologies like smartphone applications, GPS-enabled distance tracking, wearable sensors, pulse oximetry devices, and synchronous or asynchronous teleconsultation platforms [4,17].

Growing evidence suggests that remotely conducted 6MWTs and other walk tests can produce clinically meaningful and reproducible outcomes when standardized protocols, reliable devices, and appropriate supervision are employed [8,18]. Despite these promising developments, challenges remain before remote functional assessment can be fully integrated into routine clinical practice. Persistent issues include variability in environmental conditions across home settings [19], inconsistencies in test standardization [20], concerns regarding patient safety during unsupervised exertion, and the need for validated digital tools capable of accurately measuring distance, oxygen saturation, heart rate, and perceived exertion [21]. Variation in course length and turning frequency alters gait mechanics and distance estimates [22], while indoor versus outdoor testing and GPS signal loss can systematically bias speed and distance measurements [17,23]. Such effects highlight the central importance of agreement and protocol standardization in walking-test assessments. Ongoing research continues to refine methodological frameworks and develop comprehensive guidelines aimed at ensuring high measurement fidelity and clinical reliability in remote settings.

Although walking-based functional tests are widely used across both pulmonary and cardiac populations, the underlying physiological determinants of exercise limitation differ substantially between these groups. In CPDs, functional capacity is primarily constrained by ventilatory limitation, gas exchange abnormalities, and exertional hypoxemia [24], whereas in HF, reduced cardiac output, impaired peripheral oxygen delivery, skeletal muscle dysfunction, and autonomic dysregulation play a dominant role [25]. These pathophysiological differences have important implications for remote functional assessment, particularly regarding safety thresholds, monitoring requirements (e.g., oxygen saturation versus heart rate and rhythm), and the interpretation of test outcomes. Consequently, the validity, reliability, and clinical applicability of RaWTs may differ between pulmonary and cardiac populations and should be examined separately.

Previous systematic [8] and rapid [26] reviews on remotely administered functional tests in patients with CPDs [26], or cardiac conditions (mainly in HF) [8] included some studies for RaWTs up to 2020, with limited database searching [26] and without using the suitable risk of bias assessment tool for the type of the included studies [8]. The main objective of the previous systematic review [8] was to present the available remote functional tests without focusing on agreement, reliability and feasibility of the RaWTs; the rapid review reported the clinimetric properties without performing a formal quality assessment [26].

Despite growing evidence supporting tele-rehabilitation, the agreement, feasibility, reliability, and clinical utility of RaWTs remain insufficiently synthesized, particularly in populations with CPDs and HF. Within this context, the primary objective of this systematic review was to identify and synthesize studies evaluating the agreement with in-clinic testing, test–retest reliability, and feasibility of RaWTs for assessing functional capacity in individuals with CPDs (such as asthma, cystic fibrosis, chronic obstructive pulmonary disease, bronchiectasis, pulmonary hypertension, pulmonary fibrosis, and interstitial lung disease) and HF. Remote administration includes synchronous supervised testing through telecommunication technologies or fully unsupervised asynchronous testing using digital technologies (e.g., wearable sensors, smartphone applications, and GPS-enabled distance tracking). A secondary objective was to highlight the clinical utility of tests performed independently by patients in an unsupervised setting, without direct involvement from healthcare professionals, using telecommunication technologies or digital technologies. Establishing standardized, reliable methods is essential for identifying functional impairments and guiding interventions in clinical practice. Given the substantial physiological and gait-related differences between healthy individuals and patients with CPDs or HF [1,16], this systematic review was intentionally restricted to adult clinical populations to provide a focused and clinically meaningful synthesis.

The review is structured as follows: the Section 2 details the search strategy, eligibility criteria, study selection, data extraction, quality assessment, and data synthesis. The Results summarize the findings of the included studies, followed by a Discussion interpreting them in the context of existing literature, and subsections outlining real-world implementation and disease-specific considerations, methodological quality considerations, limitations of the study, and future directions.

## 2. Materials and Methods

### 2.1. Protocol and Registration

This systematic review was conducted according to the Preferred Reporting Items for Systematic Reviews and Meta-Analysis (PRISMA) guidelines [27]. The protocol was registered in the International Prospective Register of Systematic Reviews (PROSPERO: CRD420251180996) before the screening of search results. Ethics approval was not required.

### 2.2. Search Strategy

A comprehensive literature search was conducted using the following sources/databases: PubMed, Web of Science (WoS), CENTRAL, Scopus, and ACM Digital Library databases from inception up to October 2025. The ACM Digital Library was included to ensure comprehensive coverage of studies originating from the fields of digital health, wearable sensing, mobile health applications, telemonitoring systems, and human–computer interaction, where validation studies of remotely administered walking tests are frequently published and may not be indexed in medical databases [28]. Comprehensive and up-to-date search strategies were adopted to ensure search effectiveness. The search strategy was structured around four core concept blocks combined using Boolean operators: disease (pulmonary disease and heart failure), walking test, digital technology, and clinimetric properties (reliability, feasibility and clinical utility). The final search strategy is presented in the Supplementary Materials (Supplementary File S1).

### 2.3. Eligibility Criteria

In this review, observational and interventional studies were included if they addressed the following inclusion criteria, based on the PICOTS model (Population, type of Intervention, Comparator, Outcomes, Time factor, and Study design). In brief, study publications in English were included if they fulfilled the following criteria. Population: Adults (aged ≥18 years old), diagnosed with CPDs (such as asthma, cystic fibrosis, chronic obstructive pulmonary disease, bronchiectasis, pulmonary hypertension, pulmonary fibrosis, and interstitial lung disease) or heart failure (HF), and with no restrictions regarding sex, ethnicity, and socioeconomic background. Type of Interventions or exposures were functional walking tests, including the six-minute walking test (6MWT), three-minute walking test (3MWT), two-minute walking test (2MWT), incremental shuttle walking test (ISWT), and endurance shuttle walking test (ESWT). Participants performed the aforementioned tests outdoors/indoors at home or clinic, unsupervised or remotely supervised by physiotherapists or other healthcare professionals using telecommunication technologies and/or digital health sources (DHSs). DHSs include digital devices, mHealth Apps, and web-based platforms aiming to deliver health information, support exercise programs, monitor symptoms, and capture physiological responses during walking measurements [29,30]. In the context of walking test assessment, acceptable DHS-based measurement methods included GPS or application-derived distance estimation and wearable sensor-based algorithms (e.g., accelerometry-derived step count and distance) as implemented in the included studies.

Comorbid conditions were addressed at both the study selection and data extraction stages. Studies that included mixed clinical populations were only eligible if data for CPD, or HF were reported separately. During data extraction, information on reported comorbidities was recorded when available and considered qualitatively during data synthesis, particularly in relation to feasibility, safety, and agreement outcomes. Due to heterogeneity in comorbidity reporting across studies, no quantitative adjustment was possible; therefore, comorbidities were treated as a potential source of clinical heterogeneity and risk of bias and were explicitly discussed in the interpretation of findings.

Studies that examined the agreement, reliability, feasibility, safety, and clinical utility of remotely performed walking tests were included. Agreement/validity was defined by comparing remote measurements against in-person measurements under specified conditions. Reliability is defined as the quality of a measure that produces reproducible scores on repeat administrations of a test [31]. Feasibility refers to the overall practicability of conducting the study or delivering an intervention to determine whether an intervention is suitable for further testing. The feasibility includes the following domains: implementation, demand, acceptability, and practicality [32]. In this review, feasibility was operationalized using indicators most consistently reported across the included studies, namely, the following: (i) test completion and adherence rates, (ii) acceptability and usability (e.g., patient-reported ease of use or willingness to repeat the test), (iii) occurrence of technical or procedural issues during test administration, and (iv) safety-related indicators, including the reporting of adverse events. Feasibility outcomes were summarized narratively due to heterogeneity in definitions, measurement approaches, and reporting formats across studies. Safety was assessed by the documentation of adverse events during functional assessment. Adverse events were defined as unintended physical injury resulting from or contributed to by study participation. Clinical utility refers to the practical usefulness of the test or practice, specifically how its results lead to beneficial actions (e.g., treatments) that improve a patient’s health outcomes, such as reducing mortality, morbidity, or disability [33].

Studies not written in English, systematic reviews and meta-analyses, narrative reviews, book reviews, book chapters, dissertations, theses, commentaries, editorials, opinion articles, gray literature, guidelines, protocols, case reports, conference abstracts, and studies that applied tele-rehabilitation were excluded. Tele-rehabilitation studies were excluded unless the walking test itself was administered remotely. This approach ensured that the analytic sample reflected the study objective of evaluating remotely delivered walk test outcomes. Furthermore, studies with other cardiac diseases and mixed samples were excluded if data were not reported and presented separately according to the disease.

### 2.4. Study Selection

A multistage process was conducted for study selection. Firstly, all retrieved studies were imported into the Rayyan software package (https://rayyan.ai/), and duplicate studies were removed through the program. Screening decisions were standardized through predefined eligibility criteria and shared decision rules agreed upon by all reviewers before screening [27]. Two researchers (M.I., E.Ka) independently screened all potential titles and abstracts appearing to meet the inclusion/exclusion criteria. Those not meeting the eligibility criteria were removed. A full-text screening of the remaining relevant papers was conducted to determine the final eligibility with the review criteria. Discrepancies at any stage of study selection were resolved by a third independent researcher (E.A.K. for CPDs’ studies; G.P. for HF studies) for bias minimization reasons. Although formal inter-rater agreement statistics were not calculated, the same structured screening and consensus procedures were applied consistently across both disease groups to minimize the risk of inconsistency.

### 2.5. Data Extraction

Two independent researchers (M.I. and V.S. for CPDs’ studies; E.Ka and V.A. for HF studies) extracted data from eligible studies using a study-specific data extraction form with the following domains: authors, publication year, country, study design, diagnosis/population, type of walking test, technological tools used and procedures, outcome measures (reliability, validity/agreement with in-clinic testing, feasibility, safety, and clinical utility), and findings and limitations of the studies. The extraction form was developed a priori. It was piloted on a subset of included studies and refined to ensure clarity and consistency before full extraction commenced. Any discrepancies between the two reviewers were resolved through discussion, and when consensus could not be reached, a third reviewer (E.A.K) was consulted. When required data were missing or unclear, attempts were made to contact study authors for clarification. If no response was received within 2 weeks, available data were extracted as reported, and the limitations were documented accordingly.

### 2.6. Risk of Bias (Quality) Assessment

Risk of bias and methodological quality were assessed using domain-specific tools selected according to the primary aim and data contribution of each study. The methodological quality of the included studies was assessed independently by two researchers (E.A.K. and M.I. for CPDs’ studies; G.P. and E.Ka for HF studies) using the COSMIN Risk of Bias tool for the reliability studies [34], the Risk of Bias in Non-Randomized Studies of Interventions (ROBINS-I) tool [35] for non-randomized studies, and the Quality in Prognosis Studies (QUIPS) tool for the prognostic studies [36]. A third independent researcher (VS for CPDs’ studies; VA for HF studies) intervened only in cases of discrepancies between the two primary assessors.

Τhe COSMIN risk of bias checklist (Box 6) assesses the quality of studies on reliability, and it consists of 8 elements: 3 elements that constitute the study design, 4 elements for the statistical methods, and 1 element for other flaws in the design or the statistical methods. Each item is rated as “very good”, “adequate”, “doubtful”, or “inadequate”. The overall score is determined by taking the lowest rating of any element in the box.

The ROBINS-I tool was used to assess the risk of bias for seven domains: confounding, selection of participants, classification of interventions, deviations from intended interventions, missing data, measurement of outcomes, and selection of the reported results. Each domain item is rated as “low risk”, “moderate risk”, “serious risk”, “critical risk”, or “no information” and can be summed up in an overall risk of bias judgment.

The QUIPS tool includes the following six domains: study participation, study attrition, prognostic factor measurement, outcomes measurement, study confounding, statistical analysis and reporting. Studies were rated as “low risk of bias,” “moderate risk of bias,” or “high risk of bias”. Low risk of bias is defined when most or all domains are rated as low risk; moderate risk of bias is defined when one or more domains have moderate risk; high risk of bias is defined when one or more key domains are at high risk, or several domains show moderate risk. For each study, a single tool was required, aligning with the study’s primary objective.

### 2.7. Synthesis of Data

A narrative synthesis was conducted to synthesize evidence for the outcomes of this systematic review (agreement, reliability, feasibility, safety, and clinical utility) and the methodological characteristics of all included studies. Specifically, studies were grouped according to the level of supervision, supervised (real-time clinician oversight) versus unsupervised (self-administered at home), test environment, indoor versus outdoor settings, course design, fixed course length versus self-selected routes, and device type, smartphone (embedded sensors), wearable accelerometers, GPS-enabled devices, or mixed systems. 

Methodological differences and/or differences in the characteristics of the included studies, and the possible effects of individual study quality indicators (e.g., technological tools and methods used to identify adverse effects), study design, and study size were investigated and discussed. A quantitative synthesis (meta-analysis) was not performed due to substantial clinical and methodological heterogeneity across studies, including study designs, interventions, supervision levels, device types and algorithms, outcome measures, and reporting metrics, which precluded calculation of standardized effect sizes or I^2^; statistics.

## 3. Results

### 3.1. Search Results

**Figure 1:**
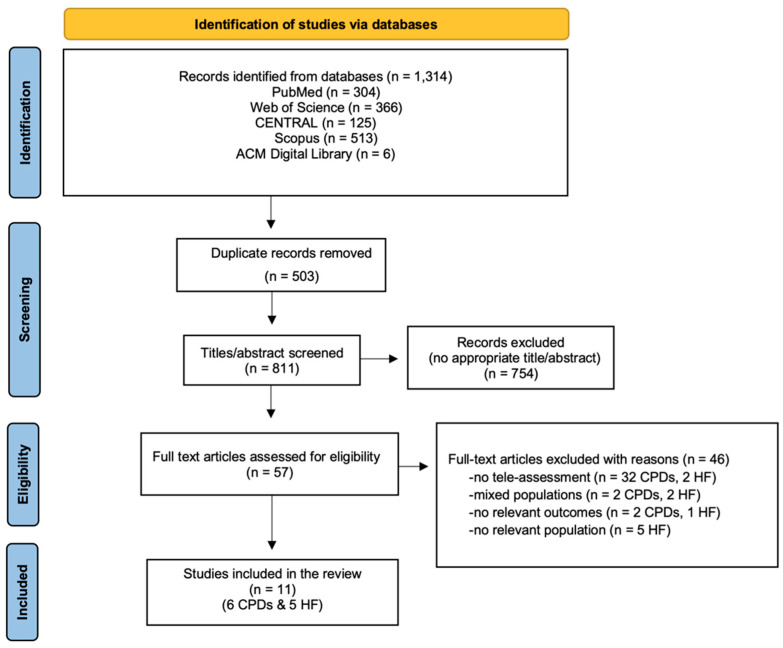
PRISMA flow diagram for the included studies.

A total of 1314 records were retrieved from the five databases, from which 503 duplicates were removed. Following title and abstract screening, 754 records were excluded. Subsequently, 57 full-text articles were assessed for eligibility, resulting in the exclusion of 46 articles with documented reasons (Figure 1). Finally, 11 studies met the inclusion criteria and were included in the systematic review (*n* = 6 for CPDs; *n* = 5 for HF).

### 3.2. Characteristics of the Included Studies

The characteristics of the included studies are presented in Table 1 and Table 2. Regarding the CPDs, six studies were included [17,20,23,37,38,39]. Of these, three were conducted in Europe [23,38,39], two were carried out in North America [17,20], and one study [37] was conducted in both aforementioned continents (Table 1). Except for one study [39], which employed a single-group pre–post intervention design, all remaining investigations were observational in nature [17,20,23,37,39]. Overall, of 179 participants included in the studies, 139 participants completed the RaWTs and were analyzed. Five studies included patients with pulmonary hypertension (PH) who completed the 6MWT [17,23,37,38,39]. One additional study included outpatients with advanced lung disease awaiting single or double lung transplantation, who performed both the 6MWT and the ISWT [20]. Only one study employed a remotely administered 6MWT (Ra6MWT) as part of a pre- and post-intervention assessment [39]. Smartphone applications were used in three studies to record walking distance when Ra6MWT was performed indoors at home [17,38] or outdoors [23], while in three studies, telecommunication platforms were utilized for remote supervision at home [20,39] or other outdoor locations [37] (Table 1). 

**Table 1:** Characteristics of the included studies for Chronic Pulmonary Diseases.

Study (Author, Year) Country	Study Design	CPDs	Sample Size (N)	Age, Years: Mean (SD) Male/Female	Remote Walking Test (Type)	Technology/Tools	Procedure	Outcome Measures/Main Findings				Limitations
								Reliability	Validity/ Agreement	Feasibility	Safety	
Salvi et al. [23] United Kingdom	Cohort prospective observational	PAH	30 (22/29 completed)	50 (16.55) 11/19	6MWT App-Supported (in clinic and outdoors)	Smartphone, Tablet Application and web interface	App-supported 6MWTs in clinic followed by monthly self-administered app-supported 6MWTs performed outdoors over 6 months.	Indoor mode: Pearson r = 0.83 (*p* < 0.0001)	Validity of Outdoor 6MWTs MD (SD): 1.80 (37.0) m ICC = 0.91	Good acceptability		Small sample; Unestimated number of outdoor tests performed under wrong conditions; The manual data quality assurance strategy was not always consistent.
LaPatra et al. [37] USA, Greece	Two-center observational pilot study	PAH CTEPH	22	51 (14) 6/16	6MWT Unsupervised remote outdoor-based assessment	Telecommunication (video or audio call)	Supervised in-person and remote 6MWT (30 m flat surface).		MD: 3 m (*p* = 0.96)	Good acceptability	1 adverse event	NR.
Lachant et al. [17] USA	Single-center, prospective observational	PAH	20	median (IQR) 59 (44–67) 4/16	6MWT (Unsupervised remote home-based)	Smartphone Application “MC10” & chest-based accelerometer	6 total 6MWTs. Tests 1 and 6: supervised in clinic. Tests 2–5: unsupervised at home.	accelerometry data (in clinic): Pearson r = 0.99 (*p* < 0.0001) accelerometer-estimated 6MWD and patient-measured 6MWD at home: Pearson r = 0.81 (*p* < 0.0001)		Feasible on a user-defined walking track	No adverse events	Small sample; Stable subjects; Heterogeneity of walking tracks at home.
Stubbs et al. [38] United Kingdom	Observational	PH	59 (24 completed 6MWT)	63 (13) 26/33	App-Supported 6MWT (unsupervised home-based)	Smartphone application: “TIMED WALK”	4 visits: 1st & 4th: Standard 6MWT in clinic. 2nd–3rd: 6MWT unsupervised at home.	Pearson r = 0.93 (*p* < 0.001)		92% acceptability	No adverse events	Small sample.
McCormack et al. [39] Ireland	Single group, pre-/post-intervention design	PH	20	49.9 (15.9) 4/16	6MWT Supervised remotely at home (*n* = 3) or a local community center (*n* = 17)	Telecommunication	10-week home-based Tele-Rehabilitation Program.			100% engagement	No adverse events	Small sample; Small number of men; Accuracy and reliability of remote monitoring NR.
Wickerson et al. [20] Canada	Prospective single-center	Advanced lung diseases	28 (24/22 completed)	64 (10) 18/4	6MWT & ISWT (home-based tele-supervised)	Telecommunication technologies (Microsoft Teams)	Supervised in clinic 6MWT and ISWT versus unsupervised remote home-based 6MWT and ISWT.		6MWT, MD (SD): 56 (57) m (LoA: −56 and 168) ISWT, MD (SD): 21 (47) m (LoA: −70 and 112)	Good Acceptability	No adverse events	Small proportion of women; Different walking tracks at home-based 6MWT; Presence of caregiver at home.

6MWD: six-minute walking distance; 6MWT: six-minute walking test; CTEPH: chronic thromboembolic pulmonary hypertension; CPDs: chronic pulmonary diseases; ISWT: incremental shuttle walk test; LoA: limits of agreement; MD: mean difference; NR: not reported; PAH: pulmonary arterial hypertension; PH: pulmonary hypertension; r: Pearson correlation coefficient; SD: standard deviation.

**Figure 2:**
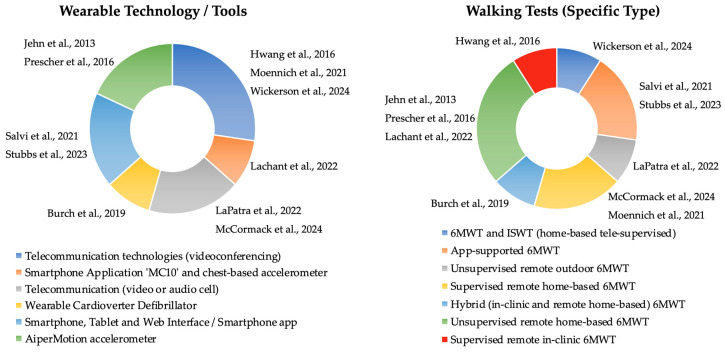
Visual presentation of the distribution of wearable tools and the specific modalities of remote walking tests utilized in the HF [4,5,40,41,42] and CPDs [17,20,23,37,38,39] studies.

Regarding the HF studies, two were conducted in Europe [4,5], one in Australia [40], one in the USA [41], and one across both European countries and the USA [42]. All studies were observational, with one being a pre-specified observational sub-study [5] of an included multicenter randomized controlled trial study [4] (Table 2). Overall, of 618 participants included in the studies, 541 participants completed the RaWTs and were analyzed. Ra6MWT was implemented in all studies. In four studies, the assessment was conducted in a home-based setting [4,5,41,42], whereas one study performed remote assessment in a clinic-based setting [40]. Videoconferencing was used in two studies [40,41], a Wearable Cardioverter-Defibrillator (WCD) was employed in one study [42], and accelerometer-based devices were used in two studies to derive six-minute walk distance from step count and gait parameters [4,5]. The distribution of the wearable tools and the specific modalities of remote walking tests utilized in the HF and the CPDs studies are illustrated in Figure 2. In total, for CPDs and HF, two studies were performed outdoors [23,37]; one study was performed in a clinic [40], and eight studies were performed at home [4,5,17,20,38,39,41,42]. No studies involving camera-based biomechanical gait analysis were identified or included.

**Table 2:** Characteristics of the included studies for heart failure.

Study (Author, Year) Country	Study Design	HF Population NYHA LVEF %: Mean (SD)	Sample Size (N)	Age, Years: Mean (SD) Male/Female	Remote Walking Test (Type)	Technology/Tools	Procedures	Outcome Measures/Main Findings					Limitations
								Reliability	Validity	Feasibility/ Acceptability	Safety	Clinical Utility	
Jehn et al. [4] Germany	Feasibility and reliability study	NYHA II–III; LVEF 27.9 (5.2)	155 (129 completed)	67.7 (10.8) 130/25	Unsupervised remote home-based 6MWT assessment	AiperMotion accelerometer	Supervised baseline in-clinic 6MWT followed by monthly unsupervised self-administered home-based 6MWTs using accelerometry over 12 months.	95% LoA: 0.96 ± 33.8 m		95% (6 months) and 84% (12 months)	1 adverse event	SF-36 correlation; NYHA discrimination; superior to self-report	Outdoor testing; Clinically stable patients.
Hwang et al. [40] Australia	Validity and reliability study	NYHA I–III; LVEF 34 (14)	17	69 (12) 15/2	Supervised remote and in-clinic 6MWT assessment	Videoconferencing	Supervised 6MWTs performed in person and remotely via real-time videoconferencing in a randomized order within a hospital setting.	Inter- and Intra-rater: ICC > 0.99	MD: 4 m (95% CI -25 to 17); ICC 0.90 (0.74–0.96)	SUS 85/100, high usability or acceptance.	2 adverse events		Small sample; selection bias; hospital environment.
Prescher et al. [5] Germany	Sub-study of a multicenter RCT	NYHA NR; LVEF 27.9 (5.2)	155 (124 event-free)	67.7 (10.8) 130/25	Unsupervised remote home-based 6MWT assessment	AiperMotion accelerometer	Supervised baseline in-clinic 6MWT followed by monthly unsupervised home-based tele-6MWTs using accelerometry over a variable follow-up period (12–21 months).	Prognosis ROC AUC for steps: 0.73 (95% CI 0.63–0.83)		43.8% (≥1 home test)		Monthly repetition did not improve prognostic value	Small sample size for prognostic analysis.
Burch et al. [42] USA, Germany, Austria	Randomized experimental validation	NYHA I–IV; LVEF 23 (7)	197	57 (12) 150/47	Hybrid (in-clinic and remote home-based) 6MWT assessment	Wearable Cardioverter Defibrillator	Clinician-guided and device-guided 6MWTs, including supervised in-clinic assessments and repeated unsupervised self-administered home-based tests.	Consistent step rate (~92 steps/min)		76% of group 2 (≥8 home tests)	No adverse events		Heterogeneity of encouraging statements and walking tracks.
Moennich et al. [41] USA	Pilot feasibility study	NYHA I-III; LVEF 41.04 (14.3)	94 (74 completed)	58.6 (11.3) 61/33	Supervised remote home-based 6MWT assessment	Video conferencing	Supervised virtual home-based 6MWTs performed at two time points and compared with supervised in-clinic 6MWTs.			78.7% both Tele-6MWTs 98.7% willing to participate via telehealth	No adverse events	Comparable to in-person testing	Small sample size; COVID impact; Limited diversity.

6MWT: Six-Minute Walking Test; AUC: Area Under the Curve; F: Female; HF: Heart Failure; ICC: Intraclass Correlation Coefficient; LoA: Limit of Agreement; LVEF: Left Ventricular Ejection Fraction; M: Male; MD: mean difference; N: number of participants; NR: Not Reported; NYHA: New York Heart Association; ROC: Receiver Operating Characteristic; SD: Standard Deviation; SF-36: patient perception of physical status; SUS: System Usability Scale; vs: Versus; WCD: Wearable Cardioverter Defibrillator.

#### 3.2.1. Validity and Agreement

The validity of unsupervised accelerometry-based assessment of Ra6MWT in HF patients was evaluated in two similar studies [4,5]. Jehn et al. [4] demonstrated 99% accuracy of remote accelerometer-based 6MWT step counts compared to hand counts in patients with HF. Furthermore, step frequency accurately reflected functional status and discriminated between New York Heart Association (NYHA) classification in patients with HF, thereby supporting the construct validity of step-based measures [4]. Similarly, Ra6MWT demonstrated good validity compared with face-to-face assessment, with no significant difference in distance (MD: 4 m, 95% CI: −25 to 17 m) and strong agreement (ICC = 0.90), supporting its use for functional assessment in patients with CHF [40]. Burch et al. [42] demonstrated that there was a statistically significant 15-step difference in median step count between the in-clinic Wearable Cardioverter Defibrillator (WCD)-guided 6MWT and the first at-home (WCD)-guided 6MWT (medians: 558 steps and 543 steps, respectively, *p* = 0.001).

In studies involving individuals with CPD, good validity of outdoor, community-based 6MWTs has been demonstrated in one study (MD: 1.8 m, SD: 37) [33]. LaPatra et al. [37] reported no difference (*p* = 0.96) in average walking distance at 6MWT between in-clinic and remotely administered walking testing (mean difference: 3 m). On the contrary, Wickerson et al. [20] indicated limited agreement between in-person and remote assessments for 6MWT and ISWT in individuals with PH. The mean difference between in-person and remote 6MWT distances was 56 ± 57 m.

#### 3.2.2. Reliability

Reliability was evaluated as a primary outcome in four CPDs studies [20,23,37,38] and three HF studies [4,40,42]. In detail, Salvi et al. [23] reported excellent reliability (r = 0.93) for an app-supported 6MWT performed outdoors or in the clinic (Table 1). However, they stated substantial variability (with differences exceeding 100 m) when testing was performed on narrow walking paths. Jehn et al. [4] demonstrated high reliability of remote accelerometer-based 6MWT measurements (step counts were shown to correlate strongly with 6MWT distance and walking speed) between repeated remote tests over 12 months (r = 0.98–0.985) in patients with HF, confirming reproducibility across time and settings. Hwang et al. [40] demonstrated excellent inter- and intra-rater reliability of the 6MWT in patients with CHF, with ICC values >0.95, supporting the reliability of tele-rehabilitation assessments for the 6MWT. Furthermore, Burch et al. [42] demonstrated that repeated weekly home-based Wearable Cardioverter Defibrillator (WCD)-guided 6MWTs showed stable step counts (*p* > 0.05), supporting the reproducibility of home-based WCD-guided testing over time.

#### 3.2.3. Feasibility and Safety

Feasibility outcomes varied across studies. In terms of adherence and completion rates, three CPDs [23,38,39] and two HF studies [4,41] incorporated feasibility as a primary outcome by noting the number of participants who completed the remote test procedures and maintained adherence throughout the study period [38,39] or by collecting physiological data (such as pulse oximetry measurements obtained during a remotely administered walking test) [31]. Jehn et al. [4] demonstrated that tele-accelerometry-based 6MWT testing is feasible in a home-based telemedicine setting, with high patient adherence and compliance over long-term follow-up. Monthly remote 6MWTs were completed by most participants, with 95% completing at least six months, and 84% completing a minimum of twelve months of monitoring. Similarly, Moennich et al. [41] showed that virtually supervised, at-home administration of the 6MWT for patients with HF is feasible (78.7% completion) and acceptable (56% comfortability with virtual visits, 60% agreement that virtual visit is as good as an in-person visit, and 98.7% willing to participate in research via telehealth).

Acceptability and usability were high in most studies. Stubbs et al. [38] reported that 24 of 29 participants (82.76%) completed the remote 6MWT, and 92% indicated that they were “very happy” or “happy” to continue performing the remote test. Only a small number of participants (*n* = 2) perceived the remote 6MWT as a more accurate reflection of their functional capacity compared with an indoor corridor test, while one participant reported difficulty completing the remote assessment due to adverse weather conditions.

Safety-related indicators, including the reporting of adverse events, were emphasized in all studies [4,17,20,37,38,39,40,41,42]. Adverse events were assessed using a combination of active surveillance [17,20,39,40,41] and passive reporting [4,17,37,38,39,42]. The safe administration of the intervention—often evidenced by the absence of reported adverse events—as well as the extent to which the protocol could be implemented as intended. Safety was assessed in five CPDs’ studies [17,20,37,38,39] by documenting the occurrence of adverse events (e.g., oxygen saturation <80%) during remote assessments. No serious adverse events were reported during the performance of distance-based functional tests, whether conducted at home, outdoors, or within local community centers. One study reported that one out of 22 participants experienced lightheadedness and tinnitus during the remote walking testing [23]. According to McCormack et al. [39], remote home-based walking testing in a 10 m corridor was feasible for patients awaiting lung transplantation, and none of the participants reported chest pain or palpitation pre- or post-training, assessed 6MWT remotely. Similarly, safety was assessed in four HF studies, indicating very few (*n* = 2) incidences of angina or onset of ventricular tachycardia (*n* = 1) during Ra6MWT, supporting the relative safety of remote functional testing in patients with HF [4,40,41,42].

Occurrence of technical or procedural issues during test administration was reported by the study of Salvi et al. [23]. Although 58% of the participants perceived the application as easy to use, several technological challenges were identified, including delays in data transmission (4 out of 18 participants) and issues with the user interface (2 out of 18 participants). Beyond technical concerns, participants highlighted weather-related constraints and privacy-related apprehensions as additional barriers to use.

#### 3.2.4. Clinical Utility

The prognostic value of the Ra6MWT was evaluated in a single study by Prescher et al. [5], which demonstrated that baseline step counts and distances recorded during the Ra6MWT were predictive of heart failure-related hospitalization or all-cause mortality. A baseline threshold of 495 steps optimally differentiated patients at risk, with high specificity (90%) but low sensitivity (26%), suggesting potential utility for ruling out short-term adverse events. Hazard ratios, calibration statistics, and multivariable prognostic modeling results were not reported and were therefore not extractable.

### 3.3. Risk of Bias for Included Studies

**Figure 3:**
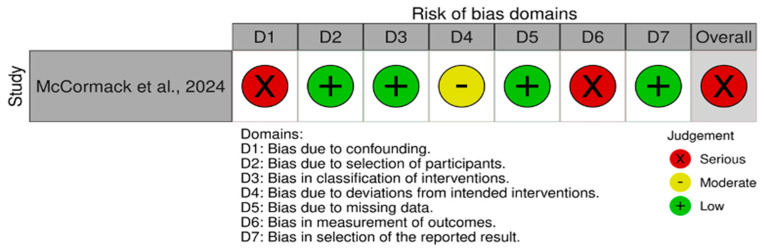
Risk of bias summary (ROBINS-I) for CPD interventional study [39].

The overall methodological quality of the CPD and HF studies (*n* = 9), using the COSMIN checklist (Box 6), ranged from very good (*n* = 2), adequate (*n* = 5), doubtful (*n* = 1), and inadequate (*n* = 1), (Table 3). The non-randomized study was evaluated using the ROBINS-I tool and was judged to be at serious risk of bias, primarily due to confounding, measurement of outcomes (Figure 3).

In addition, one prognostic study was assessed using the Quality in Prognosis Studies (QUIPS) tool, which revealed a moderate risk of bias for study participation and attrition, a low risk for prognostic factor and outcome measurement, a high risk for confounding, and a moderate risk for statistical analysis [5]. Overall, the latest study is rated as high risk of bias. Detailed results of the risk of bias assessments for each study are presented in Table 3 and Figure 3.

## 4. Discussion

### 4.1. Overview and Interpretation of Main Findings

This systematic review provides the first comprehensive synthesis of the available evidence on the agreement, reliability, feasibility, safety, and clinical utility of RaWTs in individuals with CPDs, mainly those with PH, or HF. The principal findings indicate that RaWTs, predominantly the 6MWT, are generally reliable and feasible, with high patient adherence and minimal safety concerns. However, evidence supporting agreement with in-clinic assessments and their clinical utility, particularly in terms of prognostic value and impact on clinical decision-making, remains limited.

Specifically, in the HF population, studies demonstrated that Ra6MWTs were not only feasible but also showed high levels of patient acceptance, with usability scores reaching up to 85/100 on the System Usability Scale (SUS) [40]. Furthermore, adherence rates for unsupervised home-based testing were remarkably high, with some protocols achieving 95% completion over 6 months and 84% over 12 months [4]. Similarly, in CPD populations, particularly those with PH, high levels of acceptability (up to 92%) and engagement (100%) were reported for app-supported and tele-supervised walking tests [23,38,39].

Across both disease groups, strong correlations and high intraclass correlation coefficients (ICCs) were consistently reported between repeated remote assessments or between remote and in-clinic measurements, suggesting that RaWTs can provide reproducible estimates of functional capacity when appropriate protocols and technologies are used [4,5,20,23,40]. These findings align with the growing body of literature supporting remote functional assessment as a core component of contemporary telehealth and tele-rehabilitation models [12,26]. Importantly, the present review extends previous work by focusing specifically on walking-based functional tests and by integrating a structured methodological quality assessment using COSMIN, ROBINS-I, and QUIPS frameworks. The increased reliance on remote assessment strategies following the COVID-19 pandemic has highlighted the urgent need for valid and reliable complementary tools to in-clinic testing, particularly for vulnerable cardiopulmonary populations [43]. In this context, RaWTs represent a pragmatic solution that aligns with broader digital health initiatives aimed at improving access, continuity of care, and long-term monitoring [29,30,44].

Overall, the strongest and most consistent evidence supports the feasibility and test–retest reliability of RaWTs in selected contexts, while agreement with in-clinic assessments and clinical utility remain limited, heterogeneous, and highly context-dependent.

#### 4.1.1. Agreement with In-Clinic Measurements

In HF patients, while the mean difference between real-time videoconferencing Ra6MWT and in-person testing was minimal (MD: 4 m), the limits of agreement can be broad, suggesting that while group-level agreement is high, individual variation must be considered [40]. Systematic differences between remote and center-based assessments were evident, particularly in CPD populations. In advanced lung diseases, mean differences between in-clinic and home-based 6MWT reached 56 m [20]. In line with earlier evidence, in-clinic 6MWD values tend to be greater than those obtained in home- or community-based settings, frequently exceeding the commonly used minimal important difference thresholds in some populations [19,45]. Similar discrepancies were observed in remote assessments conducted indoors or on short walking tracks, where shorter distances were consistently reported [17,20].

These differences are most likely attributable to environmental and methodological factors rather than deficiencies in the remote testing paradigm itself [19]. Shorter track lengths, increased frequency of turning, indoor space constraints, and variable walking surfaces are all known to reduce achieved walking distance and adversely affect agreement with standardized in-clinic testing [1]. Importantly, when remote outdoor assessments were performed on standardized, flat 30 m walking tracks, no significant differences in 6MWD were observed compared with center-based measurements, underscoring the critical role of environmental standardization rather than supervision status per se [37]. Similar conclusions have been reported in validation studies of automated and wearable-based 6MWT systems within cardiac rehabilitation, which demonstrate variable agreement depending on protocol standardization [3,46]. Additionally, research has shown that biomechanical factors, such as step length, significantly influence 6MWT performance in HF patients, which may be further affected by the confined spaces of home environments [22]. From a practical clinical perspective, remote testing should ideally be conducted on a flat, straight walking course of approximately 30 m with minimal turns and consistent surface conditions, following standard ATS/ERS recommendations [1], to optimize comparability with in-clinic assessments.

From a measurement science perspective, these findings emphasize the importance of clearly distinguishing between reliability (reproducibility) and agreement or validity. Importantly, correlation coefficients and ICCs primarily reflect relative reliability (i.e., the consistency of ranking between individuals) and do not quantify absolute agreement or measurement error. High correlations may coexist with substantial individual-level discrepancies. Therefore, indices of absolute agreement, such as mean differences and limits of agreement (LoA), should be interpreted alongside ICCs to better capture measurement error and the potential clinical impact of disagreement at the individual patient level.

#### 4.1.2. Reliability of Repeated Remote Measurements

Across the included studies, RaWTs demonstrated good to excellent test–retest reliability in selected contexts. Repeated home-based measurements showed strong correlations and high ICCs over extended follow-up periods (HF populations: r = 0.93–0.99; ICC > 0.95 [4,5]; (PAH populations: r = 0.83–0.93; ICC = 0.91) [23,38] indicating that remote administration does not inherently compromise measurement reproducibility.

In HF populations, tele-accelerometry-based Ra6MWTs demonstrated strong correlations across repeated measurements over prolonged follow-up periods (r = 0.98–0.99), with stable step counts, walking speed, and derived distances across serial monthly assessments extending up to 12 months [4,5]. In CPD populations, Pearson correlations between initial and subsequent remote tests were also very high (r = 0.93–0.99), indicating robust reproducibility even when the tests are unsupervised [17,38]. Similarly, wearable device-guided assessments yielded highly consistent results across repeated home-based tests, with minimal proportional variation between initial and subsequent measurements (<3%) [42].

These findings are clinically meaningful, as longitudinal monitoring of functional capacity represents a central objective of remote care and tele-rehabilitation models. From this perspective, within-patient consistency over time may be more relevant than absolute agreement with center-based values, particularly when remote assessments are used to detect functional decline, monitor disease progression, or evaluate response to intervention. Recent systematic evidence confirms that the 6MWT remains a robust tool for evaluating clinical status and prognosis in HF, with pooled ICCs reaching 0.93, reinforcing its role as a primary outcome measure in both traditional and remote settings [3]. Comparable conclusions have been reported in other tele-assessment contexts, including repeated Ra6MWT measurements in patients with type 2 diabetes mellitus, where high reliability and stability across home-based assessments were observed [13].

#### 4.1.3. Feasibility (And Safety)

Feasibility outcomes were primarily operationalized using pragmatic indicators such as participant completion rates, adherence to the testing protocol, and, in some studies, brief self-reported satisfaction questions. These findings should be interpreted as indirect indicators of feasibility rather than formal assessments of usability. Acceptability and implementation fidelity were assessed using participant completion rates and adherence to study protocols. Across the included studies, approximately 85% of participants completed all prescribed RaWTs, indicating a high level of acceptability and adherence to the protocol. In the qualitative analysis of the study by Stubbs et al. [38], all participants (100%) reported being able to complete the Ra6MWT, and 92% indicated that they were “happy” or “very happy” to continue performing the test at home. Notably, 2 of 39 participants reported that the Ra6MWT better reflected their functional capacity than the indoor corridor-based walking test. Similarly, in a previous single-center cohort study, patients with CPDs considered remote step testing acceptable and equally comfortable performed at home under remote supervision, although around 50% of those required additional telephone support to set up equipment [7].

A key concern of remotely performed functional testing (either supervised or non-supervised) is participants’ safety. All the included studies reported safety protocols to mitigate potential adverse events [4,5,17,20,23,37,38,39,40,41,42]. Five studies encouraged or required the presence of an adult to support or assist participants during testing, despite remote supervision by study personnel [17,20,37,38,39]. Three studies incorporated pre-test symptom-based pre-screening questionnaires to ensure patients’ safety before remote testing [20,37,42]. During the performance of walking tests, continuous real-time monitoring of heart rate, SpO2, and electrocardiography was conducted in four studies [17,20,23,39], and vital signs, such as blood pressure, SpO_2_;, and heart rate, were measured immediately before and after remote testing sessions [20,37,38,39]. One study [41] combined remote supervision with predefined termination criteria, scripted encouragement, and continuous monitoring, while excluding patients with recent or unstable cardiovascular conditions. The TIM-HF clinical trial [4,5] employed daily ECG, blood pressure, and weight monitoring to identify possible contraindications, resulting in one serious adverse event (out of 155 patients).

Safety outcomes were evaluated through the reporting of adverse events, suggesting that RaWTs can be implemented safely under structured conditions with appropriate risk-mitigation strategies. However, additional considerations are warranted for individuals with PH or HF. These included pre-test screening to exclude higher-risk individuals, such as participants with reduced physical function [37] and frailty [47], predefined stopping criteria for symptoms (chest pain, dizziness, severe dyspnea, and oxygen desaturation), as well as to include participants with digital literacy [48]. While no adverse events were recorded in these studies, the enrolled patients were on stable PH therapy and WHO functional classification I to III or had adult companions [17,20,37,38,39]. Importantly, no adverse events were reported in studies that used smartphone- or wearable-guided protocols adapted from validated, clinic-based walking tests, supporting the feasibility of these delivery modalities [4,17,23,38,40,41,42].

Of note, in a similar age group of patients with CPDs, the evaluation of remotely supervised functional assessment reported no adverse events and was acceptable to patients, who felt equally confident and comfortable with testing conditions (4.7/5 and 4.6/5, respectively), although participants were required to have adult companions during remote testing performance [7].

All studies included participants who had access to and were able to use compatible smartphones [23,24,25,26,27,28,29,30,31,32,33,34,35,36,37,38]. The broader applicability of RaWTs in everyday clinical practice is closely associated with patients’ digital literacy and their access to technology [49]. In the included studies, the majority of the participants were aged between 45 and 80 years. Older adults with limited digital skills, reduced confidence in smartphones, mobile devices, and wearable devices, have various concerns that can significantly limit participation. Recent evidence [49] suggests that low digital literacy is associated with reduced acceptance and continued use of digital health interventions. Therefore, digital literacy should be considered a key factor when interpreting the applicability of RaWTs and when designing future tele-rehabilitation protocols, to ensure equitable access and reliable implementation across diverse age-related populations.

#### 4.1.4. Clinical Utility

Preliminary evidence suggests prognostic value for hospitalization due to HF or all-cause mortality with prognostic performance comparable to that of the conventional 6MWT [5]. Although the included studies for CPD populations have not clearly established the clinical utility of remote assessment, the application-based 6MWT appears to enable repeated evaluation of pharmacological treatment response, whereas accelerometer-derived measurements during Ra6MWT revealed atrial tachyarrhythmia in PH populations [17,23].

### 4.2. Real-World Implementation Barriers and Disease-Specific Considerations

Despite encouraging findings regarding feasibility, the real-world implementation of RaWTs remains challenged by several practical barriers. Limited access to compatible digital devices, variable levels of digital literacy, unreliable internet connectivity, and the need for caregiver presence were recurrent issues, particularly among older and more clinically complex patients [13,46]. Environmental constraints, such as short indoor walking tracks, uneven outdoor surfaces, weather conditions, and privacy concerns, further influenced test performance and agreement with center-based assessments [37].

Importantly, implementation barriers differ between pulmonary and cardiac populations. In CPDs, particularly pulmonary hypertension and advanced lung disease, safety considerations related to exertional hypoxemia necessitate close monitoring of oxygen saturation and predefined stopping criteria, potentially limiting unsupervised implementation [50]. In contrast, in HF populations, arrhythmia risk, hemodynamic instability, and fatigue-related symptom fluctuations require attention to heart rate monitoring and pacing consistency [3]. These disease-specific considerations may partly explain the observed differences in agreement and measurement variability between CPD and HF subgroups.

Overall, while both populations demonstrate high feasibility, the broader clinical applicability appears more context-dependent in CPDs than in HF, underscoring the need for population-specific protocols.

### 4.3. Methodological Quality Considerations

Notwithstanding these favorable findings, reliability outcomes should be interpreted in the context of the moderate methodological quality of several included studies. COSMIN assessments ranged from very good to inadequate, with common limitations related to test condition similarity, statistical methodology, and reporting transparency. Studies rated as doubtful or inadequate frequently involved heterogeneous testing environments or insufficient control of confounding factors, which may partially explain variability in agreement and reliability estimates.

Overall, the available evidence indicates that the strongest support for RaWTs concerns feasibility and test–retest reliability in selected contexts, while agreement with in-clinic assessments and clinical utility remain limited.

### 4.4. Limitations

Several limitations of this systematic review should be acknowledged. First, the limited number of eligible studies, together with substantial heterogeneity in study design, patient populations, technologies, and testing environments, precludes quantitative synthesis and limits the generalizability of the findings. Therefore, the pronounced methodological heterogeneity across the included studies precluded the conduct of a meta-analysis. Second, most included studies were observational in nature and exhibited moderate to high risk of bias, particularly with respect to confounding and outcome measurement, as identified through COSMIN, ROBINS-I, and QUIPS assessments [34,35,36]. Third, protocols for RaWTs varied considerably across studies, including differences in supervision level, walking track characteristics, and measurement tools, which complicates cross-study comparisons and limits conclusions regarding measurement equivalence. Furthermore, many studies were limited by small sample sizes and a lack of diversity in patient populations (other CPD populations apart from PH, populations with limited digital literacy), which may affect the robustness of the findings. In addition, the inclusion criteria were deliberately restricted to adult patients. Pediatric or mixed-age populations differ substantially from adults in physiological responses and reference values for walking tests. While studies conducted in healthy individuals may provide useful methodological insights (e.g., regarding data processing pipelines or technical validation procedures) [51], they do not adequately reflect issues of feasibility, safety, and clinical applicability in cardiopulmonary disease populations. Fourth, although reliability and feasibility were frequently examined in patients with PH, evidence regarding validity, responsiveness, and clinical utility remains sparse, particularly in CPD populations. Additionally, we did not systematically synthesize detailed experimental setup characteristics across studies, such as device specifications (e.g., type and sampling rate). These factors are essential to clarify methodological heterogeneity and design choices in remote-monitoring walking assessments [51]. Although we extracted and summarized all available information, several studies did not comprehensively describe aspects related to protocol standardization, feasibility considerations, safety procedures, or clinical implementation. This limited reporting restricts the possibility of fully comparing design approaches and drawing definitive conclusions about optimal configurations. The manual data quality assurance strategies used in some app-supported studies were not always consistent, potentially introducing measurement error. Restricting eligibility to English-language publications may have introduced language bias and excluded relevant evidence, such as studies in Spanish or French. This limitation is partly mitigated by the comprehensive search strategy and the use of five international databases that index high-quality studies. Finally, the exclusion of gray literature may have contributed to publication bias, potentially leading to an overestimation of positive findings.

### 4.5. Future Directions

Future research should prioritize well-designed prospective studies and randomized controlled trials that directly compare remote and center-based walking tests using standardized and clearly defined protocols. Particular emphasis should be placed on establishing minimum environmental and technological requirements, including track length, surface characteristics, sensor accuracy, and acceptable levels of supervision, to improve measurement comparability and fidelity. Therefore, future studies should aim to standardize RaWT protocols, particularly with respect to (i) walking track characteristics, (ii) supervision mode, and (iii) the devices or algorithms employed, including the systematic implementation of data quality checks across settings. Innovative technologies that accurately detect abnormal gait patterns can also be applied to patients with gait abnormalities for remote walking monitoring [51].

Importantly, future studies must move beyond assessments of reliability and feasibility to address clinical utility, including responsiveness to intervention, prognostic value, and impact on clinical decision-making and patient-centered outcomes. In this context, the integration of RaWTs into multicomponent tele-rehabilitation pathways, supported by wearable sensors, automated data analytics, and clinical alert systems, represents a promising avenue for personalized and scalable care [10,21,43].

Finally, the development of consensus-based guidelines for RaWTs analogous to existing ATS/ERS recommendations for center-based testing, is essential to ensure measurement fidelity, enhance reproducibility, and facilitate broader clinical adoption in both research and routine clinical practice.

## 5. Conclusions

In conclusion, current evidence primarily supports the feasibility and test–retest reliability of RaWTs in selected cardiopulmonary populations. In particular, they demonstrate high patient adherence and minimal safety concerns when implemented under structured protocols and appropriate risk-mitigation strategies.

However, agreement with center-based assessments remains variable and strongly influenced by environmental conditions, testing setup, and disease-specific characteristics. Differences in pathophysiology between CPDs and HF appear to affect both measurement agreement and implementation requirements, underscoring the need for population-specific protocols and cautious interpretation of remotely derived outcomes.

At present, RaWTs should be considered complementary tools rather than direct substitutes for standardized in-clinic functional assessments. Remote testing may be unsuitable for higher-risk patients or in constrained indoor environments that preclude standardized track length and real-time supervision.

Overall, the strongest and most consistent evidence supports the use of RaWTs for longitudinal, within-individual monitoring of functional capacity, which represents a central objective of contemporary telehealth and tele-rehabilitation models. Evidence for agreement with in-clinic testing and for clinical utility, particularly in terms of prognostic value and impact on clinical decision-making, remains limited and context-dependent.

Further high-quality, prospective studies are required to establish responsiveness, prognostic significance, and clinically meaningful thresholds for change, as well as to inform the development of consensus-based guidelines for the safe and standardized implementation of remotely administered walking tests in routine clinical practice.

## Figures and Tables

**Table 3:** The risk of bias of the included studies, as per the COSMIN and the QUIPS risk of bias tools.

COSMIN Risk of Bias	Were Patients Stable in the Interim Period on the Construct to Be Measured?	Was the Time Interval Appropriate?	Were the Test Conditions Similar for the Measurements? for e.g., Type of Administration, Environment, Instructions	For Continuous Scores: Was an Intraclass Correlation Coefficient (ICC) Calculated?	Were There Any Other Important Flaws in the Design or Statistical Methods of the Study?	Overall Methodological Quality
Box 6 Reliability						
Jehn et al. [4]	Very good	Very good	Very good	Adequate	Very good	Adequate
Hwang et al. [40]	Very good	Very good	Very good	Very good	Very good	Very good
Burch et al. [42]	Very good	Very good	Inadequate	Doubtful	Very good	Inadequate
Moennich et al. [41]	Very good	Very good	Very good	Very good	Very good	Very good
Lachant et al. [17]	Very good	Very good	Adequate	Adequate	Very good	Adequate
LaPatra et al. [37]	Very good	Very good	Very good	Adequate	Very good	Adequate
Salvi et al. [23]	Adequate	Very good	Adequate	Very good	Very good	Adequate
Stubbs et al. [38]	Very good	Very good	Adequate	Adequate	Doubtful	Doubtful
Wickerson et al. [20]	Very good	Very good	Adequate	Adequate	Very good	Adequate
**QUIPS Tool** **Risk of Bias**	**Study Participation**	**Study Attrition**	**Prognostic Factor Measurement**	**Outcome Measurement**	**Confounding**	**Statistical Analysis**
**Prognostic**						
Prescher et al. [5]	Moderate	Moderate	Low	Low	High	Moderate

## Data Availability

No new data were created or analyzed in this study.

## Supplementary Materials

The following supporting information can be downloaded at: https://www.mdpi.com/article/10.3390/healthcare14050576/s1. File S1: Search strategy for each database.

## Abbreviations

The following abbreviations are used in this manuscript:

2MWT	Two-Minute Walking Test
3MWT	Three-Minute Walking Test
6MWT	Six-Minute Walking Test
CPDs	Chronic Pulmonary Diseases
ESWT	Endurance Shuttle Walking Test
HF	Heart Failure
ICCs	Intraclass Correlation Coefficients
ISWT	Incremental Shuttle Walking Test
NYHA	New York Heart Association
PH	Pulmonary Hypertension
QUIPS	Quality in Prognosis Studies
RaWTs	Remotely Administered Walking Tests
Ra6MWT	Remotely Administered Six-Minute Walking Test
ROBINS-I	Risk of Bias in Non-Randomized Studies—of Interventions
SUS	System Usability Scale
WCD	Wearable Cardioverter Defibrillator
WOS	Web of Science

## References

[1] Holland A.E., Spruit M.A., Troosters T., Puhan M.A., Pepin V., Saey D., McCormack M.C., Carlin B.W., Sciurba F.C., Pitta F. (2014). An official European Respiratory Society/American Thoracic Society technical standard: Field walking tests in chronic respiratory disease. Eur. Respir. J..

[2] Zamboti C.L., Pimpão H.A., Bertin L.D., Krinski G.G., Garcia T., Dos Santos Filho S.L.S., Cavalheri V., Pitta F., Camillo C.A. (2024). Functional Measures in Non-COPD Chronic Respiratory Diseases: A Systematic Review. J. Clin. Med..

[3] Pepera G., Antoniou V., Karagianni E., Batalik L., Su J.J. (2025). Validity and Reliability of the Six-Minute Walking Test Compared to Cardiopulmonary Exercise Test in Individuals with Heart Failure: Systematic Review and Meta-Analysis. J. Clin. Med..

[4] Jehn M., Prescher S., Koehler K., von Haehling S., Winkler S., Deckwart O., Honold M., Sechtem U., Baumann G., Halle M. (2013). Tele-accelerometry as a novel technique for assessing functional status in patients with heart failure: Feasibility, reliability and patient safety. Int. J. Cardiol..

[5] Prescher S., Schoebel C., Koehler K., Deckwart O., Wellge B., Honold M., Hartmann O., Winkler S., Koehler F. (2016). Prognostic value of serial six-minute walk tests using tele-accelerometry in patients with chronic heart failure: A pre-specified sub-study of the TIM-HF-Trial. Eur. J. Prev. Cardiol..

[6] Gobburi R.K., Olawade D.B., Olatunji G.D., Kokori E., Aderinto N., David-Olawade A.C. (2025). Telemedicine use in rural areas of the United Kingdom to improve access to healthcare facilities: A review of current evidence. Inform. Health.

[7] Cox N.S., Dal Corso S., Burge A.T., Bondarenko J., Perryman J., Holland A.E. (2025). Remote assessment of exercise capacity in adults with chronic respiratory disease: Safety, reliability and acceptability. Chronic Respir. Dis..

[8] Hwang R., Fan T., Bowe F., Louis M., Bertram M., Morris N.R., Adsett J. (2022). Home-based and remote functional exercise testing in cardiac conditions, during the covid-19 pandemic and beyond: A systematic review. Physiotherapy.

[9] Raidou V., Andreadou S., Christakou A., Kortianou E. (2023). Digital Health Care in Chronic Respiratory Diseases during and beyond the COVID-19 pandemic. A critical review. Med. Res. Arch..

[10] Antoniou V., Davos C.H., Kapreli E., Batalik L., Panagiotakos D.B., Pepera G. (2022). Effectiveness of Home-Based Cardiac Rehabilitation, Using Wearable Sensors, as a Multicomponent, Cutting-Edge Intervention: A Systematic Review and Meta-Analysis. J. Clin. Med..

[11] Brigo E., Rintala A., Kossi O., Verwaest F., Vanhoof O., Feys P., Bonnechère B. (2022). Using Telehealth to Guarantee the Continuity of Rehabilitation during the COVID-19 Pandemic: A Systematic Review. Int. J. Environ. Res. Public Health.

[12] Mavronasou A., Asimakos A., Vasilopoulos A., Katsaounou P., Kortianou E. (2024). Remote administration of the short physical performance battery, the 1-minute sit to stand, and the Chester step test in post-COVID-19 patients after hospitalization: Establishing inter-reliability and agreement with the face-to-face assessment. Disabil. Rehabil..

[13] Pepera G., Karanasiou E., Blioumpa C., Antoniou V., Kalatzis K., Lanaras L., Batalik L. (2023). Tele-Assessment of Functional Capacity through the Six-Minute Walk Test in Patients with Diabetes Mellitus Type 2: Validity and Reliability of Repeated Measurements. Sensors.

[14] Borges do Nascimento I.J., Abdulazeem H., Vasanthan L.T., Martinez E.Z., Zucoloto M.L., Østengaard L., Azzopardi-Muscat N., Zapata T., Novillo-Ortiz D. (2023). Barriers and facilitators to utilizing digital health technologies by healthcare professionals. npj Digit. Med..

[15] Shah A.J., Althobiani M.A., Saigal A., Ogbonnaya C.E., Hurst J.R., Mandal S. (2023). Wearable technology interventions in patients with chronic obstructive pulmonary disease: A systematic review and meta-analysis. npj Digit. Med..

[16] Piepoli M.F., Spoletini I., Rosano G. (2019). Monitoring functional capacity in heart failure. Eur. Heart J. Suppl..

[17] Lachant D., Kennedy E., Derenze B., Light A., Lachant M., White R.J. (2022). Cardiac Effort to Compare Clinic and Remote 6-Minute Walk Testing in Pulmonary Arterial Hypertension. Chest.

[18] Pires I.M., Denysyuk H.V., Villasana M.V., Sá J., Marques D.L., Morgado J.F., Albuquerque C., Zdravevski E. (2022). Development Technologies for the Monitoring of Six-Minute Walk Test: A Systematic Review. Sensors.

[19] Holland A.E., Rasekaba T., Fiore J.F., Burge A.T., Lee A.L. (2015). The 6-minute walk distance cannot be accurately assessed at home in people with COPD. Disabil. Rehabil..

[20] Wickerson L.M., de Paula Ferreira M., Rozenberg D., Mathur S., Singer L.G. (2024). In-Person Versus Remote 6-Minute Walk and Incremental Shuttle Walk Distances in Advanced Lung Disease. Respir. Care.

[21] Su J.J., Chan M.H.S., Ghisi G.L.M., Kwan R.Y.C., Wong A.K.C., Lin R., Yeung J.W.F., He Q., Pepera G., Batalik L. (2025). Real-World Mobile Health Implementation and Patient Safety: Multicenter Qualitative Study. J. Med. Internet Res..

[22] Pepera G.K., Sandercock G.R., Sloan R., Cleland J.J., Ingle L., Clark A.L. (2012). Influence of step length on 6-minute walk test performance in patients with chronic heart failure. Physiotherapy.

[23] Salvi D., Poffley E., Tarassenko L., Orchard E. (2021). App-Based Versus Standard Six-Minute Walk Test in Pulmonary Hypertension: Mixed Methods Study. JMIR Mhealth Uhealth.

[24] Kortianou E.A., Nasis I.G., Vogiatzis I. (2011). Exercise strategies for chronic respiratory diseases. Minerva Pneumol..

[25] Del Buono M.G., Arena R., Borlaug B.A., Carbone S., Canada J.M., Kirkman D.L., Garten R., Rodriguez-Miguelez P., Guazzi M., Lavie C.J. (2019). Exercise Intolerance in Patients With Heart Failure: JACC State-of-the-Art Review. J. Am. Coll. Cardiol..

[26] Holland A.E., Malaguti C., Hoffman M., Lahham A., Burge A.T., Dowman L., May A.K., Bondarenko J., Graco M., Tikellis G. (2020). Home-based or remote exercise testing in chronic respiratory disease, during the COVID-19 pandemic and beyond: A rapid review. Chronic Respir. Dis..

[27] Page M.J., McKenzie J.E., Bossuyt P.M., Boutron I., Hoffmann T.C., Mulrow C.D., Shamseer L., Tetzlaff J.M., Akl E.A., Brennan S.E. (2021). The PRISMA 2020 statement: An updated guideline for reporting systematic reviews. BMJ.

[28] AlQudah A.A., Al-Emran M., Shaalan K. (2021). Technology Acceptance in Healthcare: A Systematic Review. Appl. Sci..

[29] Dunn J., Coravos A., Fanarjian M., Ginsburg G.S., Steinhubl S.R. (2024). Remote digital health technologies for improving the care of people with respiratory disorders. Lancet Digit. Health.

[30] Gray R., Indraratna P., Lovell N., Ooi S.Y. (2022). Digital health technology in the prevention of heart failure and coronary artery disease. Cardiovasc. Digit. Health J..

[31] Batterham A.M., George K.P. (2023). Reliability in evidence-based clinical practice: A primer for allied health professionals. Phys. Ther. Sport..

[32] Bowen D.J., Kreuter M., Spring B., Cofta-Woerpel L., Linnan L., Weiner D., Bakken S., Kaplan C.P., Squiers L., Fabrizio C. (2009). How we design feasibility studies. Am. J. Prev. Med..

[33] Bowling F., Badrick T. (2023). Methods for determining clinical utility. Clin. Biochem..

[34] Mokkink L.B., Boers M., van der Vleuten C.P.M., Bouter L.M., Alonso J., Patrick D.L., de Vet H.C.W., Terwee C.B. (2020). COSMIN Risk of Bias tool to assess the quality of studies on reliability or measurement error of outcome measurement instruments: A Delphi study. BMC Med. Res. Methodol..

[35] Sterne J.A., Hernán M.A., Reeves B.C., Savović J., Berkman N.D., Viswanathan M., Henry D., Altman D.G., Ansari M.T., Boutron I. (2016). ROBINS-I: A tool for assessing risk of bias in non-randomised studies of interventions. BMJ.

[36] Hayden J.A., van der Windt D.A., Cartwright J.L., Côté P., Bombardier C. (2013). Assessing bias in studies of prognostic factors. Ann. Intern. Med..

[37] LaPatra T., Baird G.L., Goodman R., Pinder D., Gaffney M., Klinger J.R., Palevsky H.I., Fritz J., Mullin C.J., Mazurek J.A. (2022). Remote 6-Minute-Walk Testing in Patients with Pulmonary Hypertension: A Pilot Study. Am. J. Respir. Crit. Care Med..

[38] Stubbs H., Lua S., Ingram J., Jani B.D., Brewis M., Church C., Johnson M. (2023). Remote exercise testing in pulmonary hypertension (PHRET). Pulm. Circ..

[39] McCormack C., Kehoe B., Cullivan S., McCaffrey N., Gaine S., McCullagh B., McCarren A., Hardcastle S.J., Moyna N.M. (2024). Safety, feasibility and effectiveness of the remotely delivered Pulmonary Hypertension and Home-Based (PHAHB) physical activity intervention. ERJ Open Res..

[40] Hwang R., Mandrusiak A., Morris N.R., Peters R., Korczyk D., Russell T. (2017). Assessing functional exercise capacity using telehealth: Is it valid and reliable in patients with chronic heart failure?. J. Telemed. Telecare.

[41] Moennich L.A., Bittel B., Estep J.D. (2021). Virtual visits to optimize research trial offerings to heart failure patients. Cardiovasc Digit. Health J..

[42] Burch A.E., Scherr D., Rieth A., Griffin J., Bianco N.R., Odeneg T., Sears S.F. (2020). Wearable Cardioverter Defibrillator-Guided 6-Min Walk Test Performed at Home Is Accurate and Reliable: Results Of The Trends Study. J. Cardiopulm. Rehabil. Prev..

[43] Stefanakis M., Batalik L., Antoniou V., Pepera G. (2022). Safety of home-based cardiac rehabilitation: A systematic review. Heart Lung.

[44] Neubeck L., Hansen T., Jaarsma T., Klompstra L., Gallagher R. (2020). Delivering healthcare remotely to cardiovascular patients during COVID-19: A rapid review of the evidence. Eur. J. Cardiovasc. Nurs..

[45] Brooks D., Solway S., Weinacht K., Wang D., Thomas S. (2003). Comparison between an indoor and an outdoor six-minute walk test among individuals with COPD. Arch. Phys. Med. Rehabil..

[46] Nevelikova M., Dosbaba F., Pepera G., Felsoci M., Batalikova K., Su J.J., Batalik L. (2023). Validity and reliability of automated treadmill six-minute walk test in patients entering exercise-based cardiac rehabilitation. Ann. Med..

[47] Maddocks M., Brighton L.J., Alison J.A., Ter Beek L., Bhatt S.P., Brummel N.E., Burtin C., Cesari M., Evans R.A., Ferrante L.E. (2023). Rehabilitation for People with Respiratory Disease and Frailty: An Official American Thoracic Society Workshop Report. Ann. Am. Thorac. Soc..

[48] Zapletal A., Wells T., Russell E., Skinner M.W. (2023). On the triple exclusion of older adults during COVID-19: Technology, digital literacy and social isolation. Soc. Sci. Humanit. Open.

[49] Anisha S.A., Sen A., Ahmad B., Bain C. (2025). Exploring Acceptance of Digital Health Technologies for Managing Non-Communicable Diseases Among Older Adults: A Systematic Scoping Review. J. Med. Syst..

[50] Ghisi G.L.M., Kim W.S., Cha S., Aljehani R., Cruz M.M.A., Vanderlei L.C.M., Pepera G., Liu X., Xu Z., Maskhulia L. (2023). Women’s Cardiac Rehabilitation Barriers: Results of the International Council of Cardiovascular Prevention and Rehabilitation’s First Global Assessment. Can. J. Cardiol..

[51] Palazzo L., Suglia V., Grieco S., Buongiorno D., Pagano G., Bevilacqua V. Optimized Deep Learning-Based Pathological Gait Recognition Explored Through Network Analysis of Inertial Data. Proceedings of the 2025 IEEE Medical Measurements & Applications (MeMeA).

