# Supplementary material for: Remotely Administered Walking Tests for Assessing Functional Capacity in Patients with Chronic Pulmonary Diseases or Heart Failure: A Systematic Review of Agreement, Reliability, Feasibility and Clinical Utility
Source: Healthcare (Basel). 2026 Feb 25;14(5):576. doi: 10.3390/healthcare14050576 (PMC12984982; doi:10.3390/healthcare14050576)
Supplement: Supplementary file 1 [file healthcare-14-00576-s001.zip › healthcare-4115366-supplementary.pdf]

**APPENDIX.** Search strategy for each database

**PubMed Search Formula via NLM. Results: 302**

“Pulmonary Disease, Chronic Obstructive”[Mesh] OR “Asthma”[Mesh] OR “Cystic Fibrosis”[Mesh] OR “Bronchiectasis”[Mesh] OR “Heart Failure”[Mesh] OR “COPD”[tw] OR “chronic obstructive”[tw] OR “asthma”[tw] OR “interstitial lung”[tw] OR “pulmonary fibrosis”[tw] OR “cystic fibrosis”[tw] OR “bronchiectasis”[tw] OR “pulmonary hypertension”[tw] OR “heart failure”[tw] OR “HFpEF”[tw] OR “HFrEF”[tw] OR “interstitial lung disease”[tw] AND “Exercise Test”[Mesh] OR “Walking” [Mesh] OR “Physical Fitness”[Mesh] OR “6-minute walk test”[tw] OR “6MWT”[tw] OR “six-minute walk test”[tw] OR “3-minute walk test”[tw] OR “3MWT”[tw] OR “three-minute walk test”[tw] OR “2-minute walk test”[tw] OR “2MWT”[tw] OR “two-minute walk test”[tw] OR “incremental shuttle walk test”[tw] OR “ISWT”[tw] OR “endurance shuttle walk test”[tw] OR “ESWT”[tw] OR “exercise test*”[tw] OR “physical function”[tw] OR “functional capacity”[tw] OR “exercise capacity”[tw] OR “physical fitness”[tw] AND “Telemedicine”[Mesh] OR “Digital Technology”[Mesh] OR “Wearable Electronic Devices”[Mesh] OR “Telemedicine”[tw] OR “mhealth”[tw] OR “m-health”[tw] OR “mobile health”[tw] OR “mobile application”[tw] OR “smartphone”[tw] OR “smart phone”[tw] OR “smartphone application”[tw] OR “cellphone”[tw] OR “cellular phone”[tw] OR “telerehabilitation”[tw] OR “tele-rehabilitation”[tw] OR “virtual rehabilitation”[tw] OR “remote rehabilitation”[tw] OR “remote intervention”[tw] OR “telehealth”[tw] OR “tele-health”[tw] OR “telemonitor”[tw] OR “ehealth”[tw] OR “e-health”[tw] OR “digital health”[tw] OR “mobile technolog*”[tw] OR “website”[tw] OR “web-site”[tw] OR “web-based”[tw] OR “tele-supervised”[tw] OR “teletherapy”[tw] OR “wireless technology”[tw] OR “telemetry”[tw] OR “videoconferencing”[tw] OR “home-based rehabilitation”[tw] OR “home exercise training”[tw] OR “tele-guidance”[tw] OR “e-coach”[tw] OR “e-coaching”[tw] OR “tele-advice”[tw] OR “games”[tw] OR “tele-physiotherapy”[tw] OR “tele-intervention”[tw] OR “teleintervention”[tw] OR “tele-education”[tw] OR “teletreatment”[tw] OR “teletraining”[tw] OR “telecoaching”[tw] OR “video games”[tw] OR “serious games”[tw] OR “wearable*”[tw] OR “wearable devices”[tw] OR “activity tracker”[tw] OR “sensor-based”[tw] AND “Feasibility Studies”[Mesh] OR “Reproducibility of Results”[Mesh] OR “Observer Variation”[Mesh] OR “feasibility”[tw] OR “practicab*”[tw] OR “acceptab*”[tw] OR “usability”[tw] OR “clinical utility”[tw] OR “implementation”[tw] OR “uptake”[tw] OR “adherence”[tw] OR “safety”[tw] OR “reliability”[tw] OR “reliable”[tw] OR “reproducib*”[tw] OR “repeatab*”[tw] OR “test-retest”[tw] OR “interrater”[tw] OR “intrarater”[tw] OR “agreement”[tw] OR “intraclass correlation”[tw] OR “ICC”[tw] OR “Bland-Altman”[tw] OR “measurement error”[tw] OR “validity”[tw] OR “validat*”[tw] OR “criterion validity”[tw] OR “construct validity”[tw] OR “responsiveness”[tw] OR “patient satisfaction”[tw] OR “acceptability”[tw] OR “user experience”[tw] OR “qualitative research”[tw] OR “concurrent validity”[tw] OR “convergent validity”[tw] AND #1 AND #2 AND #3 AND #4

**Web of Science (WoS) Formula Results:366**

“Pulmonary Disease, Chronic Obstructive” OR “Asthma” OR “Cystic Fibrosis” OR “Bronchiectasis” OR “Heart Failure” OR “COPD” OR “chronic obstructive” OR “asthma” OR “interstitial lung” OR “pulmonary fibrosis” OR “cystic fibrosis” OR “bronchiectasis” OR “pulmonary hypertension” OR “heart failure” OR “HFpEF” OR “HFrEF” OR “interstitial lung disease” AND “Exercise Test” OR “Walking” OR “Physical Fitness” OR “6-minute walk test” OR “6MWT” OR “six-minute walk test” OR “3-minute walk test” OR “3MWT” OR “three-minute walk test” OR “2-minute walk test” OR “2MWT” OR “two-minute walk test” OR “incremental shuttle walk test” OR “ISWT” OR “endurance shuttle walk test” OR “ESWT” OR “exercise test*” OR “physical function” OR “functional capacity” OR “exercise capacity” OR “physical fitness” AND “Telemedicine” OR “Digital Technology” OR “Wearable Electronic Devices” OR “mhealth” OR “m-health” OR “mobile health” OR “mobile application” OR “smartphone” OR “smartphone application” OR “cellphone” OR “cellular phone” OR “telerehabilitation” OR “tele-rehabilitation” OR “virtual rehabilitation” OR “remote rehabilitation” OR “remote intervention” OR “telehealth” OR “telemonitor” OR “ehealth” OR “e-health” OR “digital health” OR “mobile technolog*” OR “website” OR “web-based” OR “tele-supervised” OR “teletherapy” OR “wireless technology” OR “telemetry” OR “videoconferencing” OR “home-based rehabilitation” OR “home exercise training” OR “tele-guidance” OR “e-coach” OR “e-coaching” OR “tele-advice” OR “games” OR “tele-physiotherapy” OR “tele-intervention” OR “teleintervention” OR “tele-education” OR “teletreatment” OR “teletraining” OR “telecoaching” OR “video games” OR “wearable*” OR “wearable devices” OR “activity tracker” OR “sensor-based” AND “Feasibility Studies” OR “Reproducibility of Results” OR “Observer Variation” OR “feasibility” OR “practicab*” OR “acceptab*” OR “usability” OR "clinical utility" OR “implementation” OR “uptake” OR “adherence” OR “safety” OR “reliability” OR “reliable” OR “reproducib*” OR “repeatab*” OR “test-retest” OR “interrater” OR “intrarater” OR “agreement” OR “intraclass correlation” OR “ICC” OR “Bland-Altman” OR “measurement error” OR “responsiveness” OR “patient satisfaction” OR “acceptability” OR “user experience” OR “sensitivity” AND #1 AND #2 AND #3 AND #4

**CENTRAL Formula Results: 125**

ID Search Hits

#1 MeSH descriptor: [Pulmonary Disease, Chronic Obstructive] explode all trees 8223

#2 MeSH descriptor: [Asthma] explode all trees 14763

#3 MeSH descriptor: [Cystic Fibrosis] explode all trees 2351

#4 MeSH descriptor: [Bronchiectasis] explode all trees 510

#5 MeSH descriptor: [Heart Failure] explode all trees 15105

#6 COPD OR “chronic obstructive” OR asthma OR “interstitial lung” OR “pulmonary fibrosis” OR “cystic fibrosis” OR bronchiectasis OR “pulmonary hypertension” OR “heart failure” OR HFpEF OR HFrEF OR “interstitial lung disease” OR “valvular heart disease” OR “congenital heart disease” 118971

#7 #1 OR #2 OR #3 OR #4 OR #5 OR #6 119312

#8 MeSH descriptor: [Exercise Test] explode all trees 11145

#9 MeSH descriptor: [Walking] explode all trees 8593
#10 MeSH descriptor: [Physical Fitness] explode all trees 5172
#11 "6-minute walk test" OR "6MWT" OR "six-minute walk test" OR "3-minute walk test" OR "3MWT" OR "three-minute walk test" OR "2-minute walk test" OR "2MWT" OR "two-minute walk test" OR "incremental shuttle walk test" OR "ISWT" OR "endurance shuttle walk test" OR "ESWT" OR "exercise test*" OR "physical function" OR "functional capacity" OR "exercise capacity" OR "physical fitness" 60643
#12 #8 OR #9 OR #10 OR #11 67243
#13 MeSH descriptor: [Telemedicine] explode all trees 5810
#14 MeSH descriptor: [Digital Technology] explode all trees 30
#15 MeSH descriptor: [Wearable Electronic Devices] explode all trees 1180
#16 Telemedicine OR mhealth OR "m-health" OR "mobile health" OR "mobile application" OR smartphone OR "smart phone" OR "smartphone application" OR cellphone OR "cellular phone" OR telerehabilitation OR "tele-rehabilitation" OR "virtual rehabilitation" OR "remote rehabilitation" OR "remote intervention" OR telehealth OR "tele-health" OR telemonitor OR ehealth OR "e-health" OR "digital health" OR "mobile technology" OR website OR "web-site" OR "web-based" OR "tele-supervised" OR teletherapy OR "wireless technology" OR telemetry OR videoconferencing OR "home-based rehabilitation" OR "home exercise training" OR "tele-guidance" OR "e-coach" OR "e-coaching" OR "tele-advice" OR games OR "tele-physiotherapy" OR "tele-intervention" OR teleintervention OR "tele-education" OR teletreatment OR teletraining OR telecoaching OR "video games" OR "serious games" OR wearable OR "wearable devices" OR "activity tracker" OR "sensor-based" 49921
#17 #13 OR #14 OR #15 OR #16 50814
#18 MeSH descriptor: [Feasibility Studies] explode all trees 11883
#19 MeSH descriptor: [Reproducibility of Results] explode all trees 16764
#20 MeSH descriptor: [Observer Variation] explode all trees 2685
#21 feasibility OR practicability OR acceptability OR usability OR "clinical utility" OR implementation OR uptake OR adherence OR safety OR reliability OR reliable OR reproducibility OR repeatability OR "test-retest" OR interrater OR intrarater OR agreement OR "intraclass correlation" OR ICC OR "Bland-Altman" OR "measurement error" OR validity OR validate OR "criterion validity" OR "construct validity" OR responsiveness OR "patient satisfaction" OR acceptability OR "user experience" OR "qualitative research" OR "concurrent validity" OR "convergent validity" 603210
#22 #18 OR #19 OR #20 OR #21 604075
#23 #7 AND #12 AND #17 AND #22 379
#24 #7 AND #12 AND #17 AND #22 in Cochrane Reviews 125

**Scopus Formula Results: 513**

( TITLE-ABS-KEY ("Pulmonary Disease, Chronic Obstructive" OR "Asthma" OR "Cystic Fibrosis" OR "Bronchiectasis" OR "Heart Failure" OR "COPD" OR "chronic obstructive" OR "asthma" OR "interstitial lung" OR "pulmonary fibrosis" OR "cystic fibrosis" OR "bronchiectasis" OR "pulmonary hypertension" OR "heart failure" OR "HFpEF" OR "HFrEF" OR "interstitial lung disease") AND TITLE-ABS-KEY ("Exercise Test" OR "Walking" OR "Physical Fitness" OR "6-minute walk test" OR "6MWT" OR "six-minute walk test" OR "3-minute walk test" OR "3MWT" OR "three-minute walk test" OR "2-minute walk test" OR "2MWT" OR "two-minute

walk test” OR “incremental shuttle walk test” OR “ISWT” OR “endurance shuttle walk test” OR “ESWT” OR “exercise test*” OR “physical function” OR “functional capacity” OR “exercise capacity” OR “physical fitness”) AND TITLE-ABS-KEY ( “Telemedicine” OR “Digital Technology” OR “Wearable Electronic Devices” OR “Telemedicine” OR “mhealth” OR “m-health” OR “mobile health” OR “mobile application” OR “smartphone” OR “smart phone” OR “smartphone application” OR “cellphone” OR “cellular phone” OR “telerehabilitation” OR “tele-rehabilitation” OR “virtual rehabilitation” OR “remote rehabilitation” OR “remote intervention” OR “telehealth” OR “tele-health” OR “telemonitor” OR “ehealth” OR “e-health” OR “digital health” OR “mobile technolog*” OR “website” OR “web-site” OR “web-based” OR “tele-supervised” OR “teletherapy” OR “wireless technology” OR “telemetry” OR “videoconferencing” OR “home-based rehabilitation” OR “home exercise training” OR “tele-guidance” OR “e-coach” OR “e-coaching” OR “tele-advice” OR “games” OR “tele-physiotherapy” OR “tele-intervention” OR “teleintervention” OR “tele-education” OR “teletreatment” OR “teletraining” OR “telecoaching” OR “video games” OR “wearable*” OR “wearable devices” OR “activity tracker” OR “sensor-based”) AND TITLE-ABS-KEY (“Feasibility Studies” OR “Reproducibility of Results” OR “Observer Variation” OR “feasibility” OR “practicab*” OR “acceptab*” OR “usability” OR “clinical utility” OR “implementation” OR “uptake” OR “adherence” OR “safety” OR “reliability” OR “reliable” OR “reproducib*” OR “repeatab*” OR “test-retest” OR “interrater” OR “intrarater” OR “agreement” OR “intraclass correlation” OR “ICC” OR “Bland-Altman” OR “measurement error” OR “responsiveness” OR “patient satisfaction” OR “acceptability” OR “user experience” OR “sensitivity”) )

**ACM Digital Library Results: 6**
[[All: “pulmonary disease, chronic obstructive”] OR [ALL: “asthma”] OR [ALL: “cystic fibrosis”] OR [ALL: “bronchiectasis”] OR[All: “heart failure”] OR [All: “copd”] OR [All: “chronic obstructive”] OR [ALL: “asthma”] OR [All: “interstitial lung”] OR [ALL: “pulmonary fibrosis”] OR [ALL: “cystic fibrosis”] OR [ALL: “bronchiectasis”] OR [ALL: “pulmonary hypertension”] OR [ALL: “heart failure”] OR[All: “hfpef”] OR [All: “hfref”] OR [All: “interstitial lung disease”]] AND [[All: “exercise test”] OR [All: “walking”] OR [All: “physical fitness”] OR [All: “6-minute walk test”] OR [All: “6mwt”] OR [All: “six-minute walk test”] OR [All: “3-minute walk test”] OR [All: “3mwt”] OR [All: “three minute walk test”] OR [All: “2-minute walk test”] OR [All: “2mwt”] OR [All: “two-minute walk test”] OR [All: “incremental shuttle walk test”] OR [All: “iswt”] OR [All: “endurance shuttle walk test”] OR [All: “eswt”] OR [All: “exercise test*”] OR [All: “physical function”] OR [All: “functional capacity”] OR [All: “exercise capacity”] OR [All: “physical fitness”]] AND [[All: “telemedicine”] OR [All: “digital technology”] OR [All: “wearable electronic devices”] OR [All: “telemedicine”] OR [All: “mhealth”] OR [All: “m-health”] OR [All: “mobile health”] OR [All: “mobile application”] OR [All: “smartphone”] OR [All: “smart phone”] OR [All: “smartphone application”] OR [All: “cellphone”] OR [All: “cellular phone”] OR [All: “telerehabilitation”] OR [All: “tele-rehabilitation”] OR [All: “virtual rehabilitation”] OR [All: “remote rehabilitation”] OR [All: “remote intervention”] OR [All: “telehealth”] OR [All: “tele-health”] OR [All: “telemonitor”] OR [All: “ehealth”] OR [All: “e-health”] OR [All: “digital health”] OR [All: “mobile technolog*”] OR [All: “website”] OR [All: “web-site”] OR [All: “web-based”] OR [All: “tele-supervised”] OR [All: “teletherapy”] OR [All:

"wireless technology"] OR [All: "telemetry"] OR [All: "videoconferencing"] OR [All: "home-based rehabilitation"] OR [All: "home exercise training"] OR [All: "tele-guidance"] OR [All: "e-coach"] OR [All: "e-coaching"] OR [All: "tele-advice"] OR [All: "games"] OR [All: "tele-physiotherapy"] OR [All: "tele-intervention"] OR [All: "teleintervention"] OR [All: "tele-education"] OR [All: "teletreatment"] OR [All: "teletraining"] OR [All: "telecoaching"] OR [All: "video games"] OR [All: "wearable*"] OR [All: "wearable devices"] OR [All: "activity tracker"] OR [All: "sensor-based"]] AND [[All: "feasibility studies"] OR [All: "reproducibility of results"] OR [All: "observer variation"] OR [All: "feasibility"] OR [All: "practicab*"] OR [All: "acceptab*"] OR [All: "usability"] OR [All: "clinical utility"] OR [All: "implementation"] OR [All: "uptake"] OR [All: "adherence"] OR [All: "safety"] OR [All: "reliability"] OR [All: "reliable"] OR [All: "reproducib*"] OR [All: "repeatab*"] OR [All: "test-retest"] OR [All: "interrater"] OR [All: "intrarater"] OR [All: "agreement"] OR [All: "intraclass correlation"] OR [All: "icc"] OR [All: "bland-altman"] OR [All: "measurement error"] OR [All: "responsiveness"] OR [All: "patient satisfaction"] OR [All: "acceptability"] OR [All: "user experience"] OR [All: "sensitivity"]]
